# Enhanced external counter pulsation therapy in patients with symptomatic and severe intracranial steno-occlusive disease: a randomized clinical trial protocol

**DOI:** 10.3389/fneur.2023.1177500

**Published:** 2023-05-30

**Authors:** Vijay K. Sharma, Anil Gopinathan, Benjamin Y. Q. Tan, Poay Huan Loh, Jennifer Hung, David Tang, Christopher Chua, Amanda C. Y. Chan, Jonathan J. Y. Ong, Amanda Chin, Mingxue Jing, Yihui Goh, Sibi Sunny, Chin Howe Keat, Zhang Ka, Shivani Pandya, Lily Y. H. Wong, Jin Tao Chen, Leonard L. L. Yeo, Bernard P. L. Chan, Hock Luen Teoh, Arvind K. Sinha

**Affiliations:** ^1^Divisin of Neurology, National University Hospital, Singapore, Singapore; ^2^Yong Loo Lin School of Medicine, National University of Singapore, Singapore, Singapore; ^3^Department of Diagnostic Imaging, National University Hospital, Singapore, Singapore; ^4^Department of Cardiology, National University Hospital, Singapore, Singapore

**Keywords:** ischemic stroke, intracranial stenosis, transcranial Doppler, enhanced external counter pulsation (EECP), cerebral vasodilatory reserve

## Abstract

Intracranial stenosis is prevalent among Asians and constitutes a common cause of cerebral ischemia. While the best medical therapy carries stroke recurrence rates in excess of 10% per year, trials with intracranial stenting have been associated with unacceptable peri-procedural ischemic events. Cerebral ischemic events are strongly related to the severity of intracranial stenosis, which is high in patients with severe intracranial stenosis with poor vasodilatory reserve. Enhanced External Counter Pulsation (EECP) therapy is known to improve myocardial perfusion by facilitating the development of collateral blood vessels in the heart. In this randomized clinical trial, we evaluate whether EECP therapy may be useful in patients with severe stenosis of intracranial internal carotid (ICA) or middle cerebral artery (MCA). The review of literature, methods of evaluation, status of currently used therapeutic approaches, and trial protocol have been presented.

**Clinical trial registration:**

ClinicalTrials.gov, Identifier: NCT03921827.

## Introduction

Atherosclerosis of cerebral vessels is a common cause of ischemic stroke. Among Asian stroke patients, intracranial atherosclerotic disease (ICAD) is more common and accounts for ~30–50% of all strokes as compared to only 5–10% of strokes in their Caucasian counterparts (1–3). This difference is believed to be most likely related to genetic susceptibility, different lifestyles, and a risk factor profile (2, 4, 5).

After an acute ischemic stroke (AIS) or transient ischemic attack (TIA), the risk of recurrent cerebral ischemia within 90 days is ~10% (6–8). Among patients with significant ICAD, this risk increases to more than 20% in 1 year despite the best medical treatment (9). The rate of recurrence of cerebral ischemia in ICAD is determined by the degree of stenosis as well as the presence of collaterals. In warfarin–aspirin symptomatic intracranial disease (WASID) study, patients with more than 70% stenosis of an intracranial artery had a greater risk of a recurrent event when compared to those with lesser (50–69%) degree of stenosis (9). Interestingly, patients with more than 70% intracranial stenosis with good collaterals carry a lower risk for subsequent cerebral ischemic events (10, 11).

The two main mechanisms of ischemic stroke in ICAD patients are thromboembolism and cerebral hemodynamic insufficiency (12–15). While transcranial Doppler (TCD) monitoring can establish the thrombo-embolic phenomenon by identifying the spontaneous micro-embolic signals in the arterial segments distal to the site of stenosis (16–18) and help in planning appropriate antithrombotic treatment (19), assessment of cerebral hemodynamic insufficiency remains a complex issue and may serve as a potential target for improving the outcomes in patients with severe intracranial stenosis.

## Current treatment options for ICAD

### Anti-platelet agents

Anti-platelet agents are the mainstay of secondary prevention of stroke (20, 21). Earlier studies suggested warfarin to be better than aspirin for ICAD (22). However, the WASID trial did not show any significant difference in stroke recurrence between warfarin and aspirin. Furthermore, aspirin was found to be safer as compared to warfarin (23).

The use of short-term double antiplatelet therapy (DAPT) is effective in reducing stroke recurrence risk in patients with minor stroke or TIA (22, 24). In a TCD study, patients treated with short-term clopidogrel plus aspirin demonstrated a significant reduction in the number of spontaneous emboli, as compared to aspirin alone, in the relevant intracranial artery in patients with acute symptomatic cerebral or carotid artery stenosis (CLAIR) (25). The use of DAPT in intracranial stenosis was also supported by the stenting vs. aggressive medical therapy for intracranial arterial stenosis (SAMMPRIS) trial (26).

### Surgical treatment

Extracranial to intracranial (EC-IC) bypass has been compared with medical management in patients with intracranial stenosis, and it did not lower the risk of recurrent ischemic stroke. Furthermore, it was associated with worse outcomes as compared to medical management (27, 28). However, in a recent prospective observational study, we demonstrated that superficial temporal artery-middle cerebral artery (STA-MCA) bypass surgery in carefully selected patients with severe intracranial stenosis of the intracranial internal carotid artery (ICA) or middle cerebral artery (MCA) results in significant improvement in cerebral hemodynamic parameters as well as led to absolute reduction (32% during 34 months follow up) in stroke recurrence (29). Interestingly, the benefits in stroke recurrence persisted during an extended follow-up of more than 6 years (30).

### Endovascular treatment

The self-expanding Wingspan stent was approved for medically refractory patients with 50–99% stenosis of a major intracranial artery (31). In total, two multicenter registries showed the feasibility of angioplasty with a Wingspan stent (32, 33). However, the SAMMPRIS trial was stopped prematurely as the angioplasty and stenting group showed 14.7% event rate (stroke or death) at 30 days as compared to the medical management arm (5.8% event rate) (26). Similarly the Vitesse Intracranial Stent Study for Ischemic Stroke Therapy (VISSIT) trial was terminated prematurely due to a significant increase in peri-procedural complications (34). However, a Chinese trial found that intracranial stenting could be a safe and efficient treatment for carefully selected patients with MCA stenosis (35). Similarly improved safety of intracranial stenting was reported in the Wingspan Stent System Post Market Surveillance (WEAVE) trial (36). Recent studies with drug-eluting stents have shown promise in satisfactory recanalization, coupled with lesser chances of restenosis as well as other peri-procedural complications (37). With the advent of rapid improvements in stent technology, stenting for ICAD is expected to become a standard of care in future if safety and efficacy could be established in adequately powered randomized clinical trials.

### Enhanced external counter pulsation therapy

**Figure 1:**
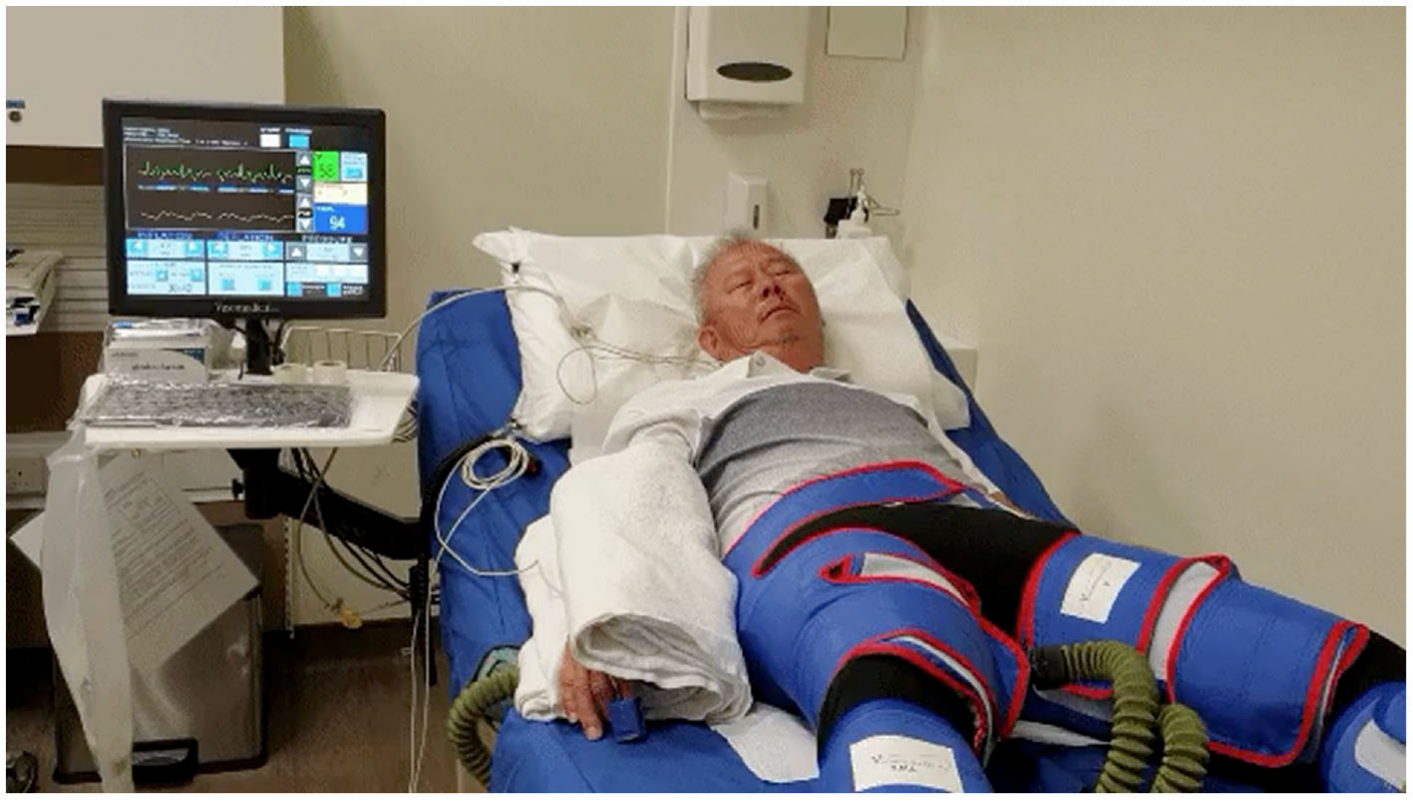
Set-up for enhanced external counter pulsation (EECP) therapy equipment. This figure shows a patient with cuffs over both lower limbs as well the monitor, which shows patient ECG as well as continuous finger plethysmography.

EECP has been used as a non-invasive therapy for treating patients with angina, congestive heart failure, acute myocardial infarction, and cardiogenic shock (38–40). It uses ECG-triggered pressure application during ventricular diastole delivered by air-filled cuffs placed around the lower limbs to improve systemic hemodynamics (Figure 1). EECP leads to a nearly 25% improvement in arterial flow volume in carotid, renal, and hepatic arteries along with a 20–40% improvement in coronary artery blood flow and a 12% increase in left ventricular (LV) stroke volume (41). EECP improves cardiac functional class, decreased anginal episodes, and reduced nitroglycerin use in patients with refractory angina (42). Although it is non-invasive, EECP produces similar hemodynamic effects as the intra-aortic balloon pump (43).

**Figure 2:**
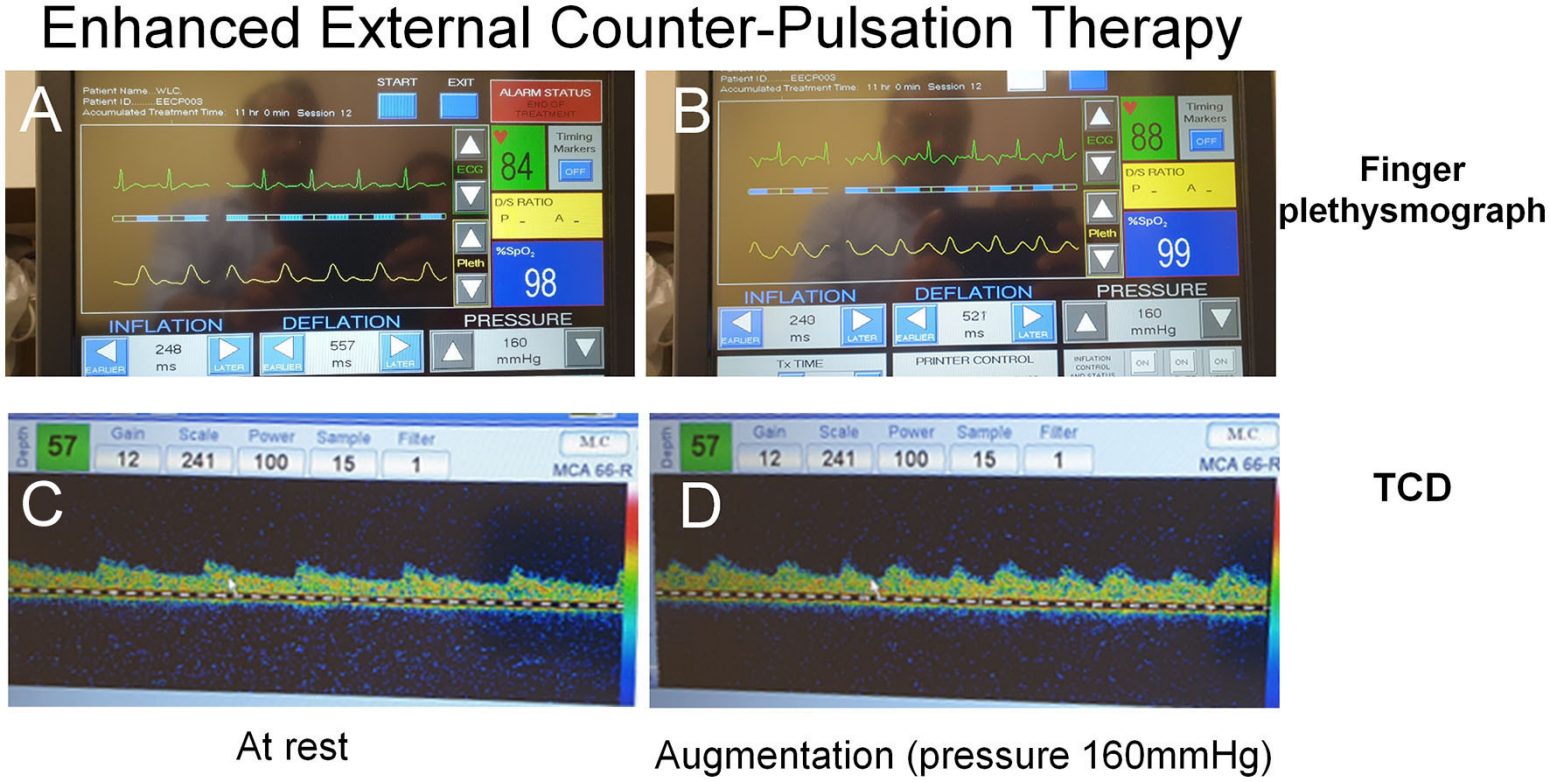
Changes in finger plethysmography and transcranial Doppler spectra from middle cerebral artery during EECP therapy. **(A)** Shows the ECG and finger plethysmography at rest while the change in the waveform after applying an augmentation pressure of 160 mmHg is shown in **(B)**. Note the second peak during diastole. Corresponding changes on TCD at rest and during EECP therapy are shown in **(C, D)**, respectively.

EECP may help in the recovery of stroke patients with large artery occlusive disease (44). The hemodynamic alterations induced during EECP therapy may be monitored in real time by continuous finger plethysmography as well as TCD (Figure 2). In a Hong Kong study of 155 patients with ICAD, EECP treatment was found to be a significant factor for predictor of good outcomes at 3 months (45). In another preliminary study, EECP performed on healthy volunteers induced marked changes in cerebral arterial waveforms and augmented peak diastolic and mean MCA flow velocities on TCD (46). Given its low-risk complication profile, EECP may offer an alternative therapeutic approach for improving cerebral hemodynamics in patients with severe ICAD.

## Aims of the study

### Primary aim

The study aimed to evaluate whether EECP therapy that would lead to an improvement in cerebral vasodilatory reserve (CVR) in patients with severe and recently symptomatic stenosis of intracranial carotid (ICA) or middle cerebral artery (MCA).

### Secondary aims

1. To evaluate the impact of EECP on the recurrence of a cerebral ischemic event in patients with severe and recently symptomatic stenosis of ICA or MCA.
2. To evaluate the impact of EECP on neurocognitive performance in patients with severe and recently symptomatic stenosis of ICA or MCA.

## Methods and analysis

### Design

**Figure 3:**
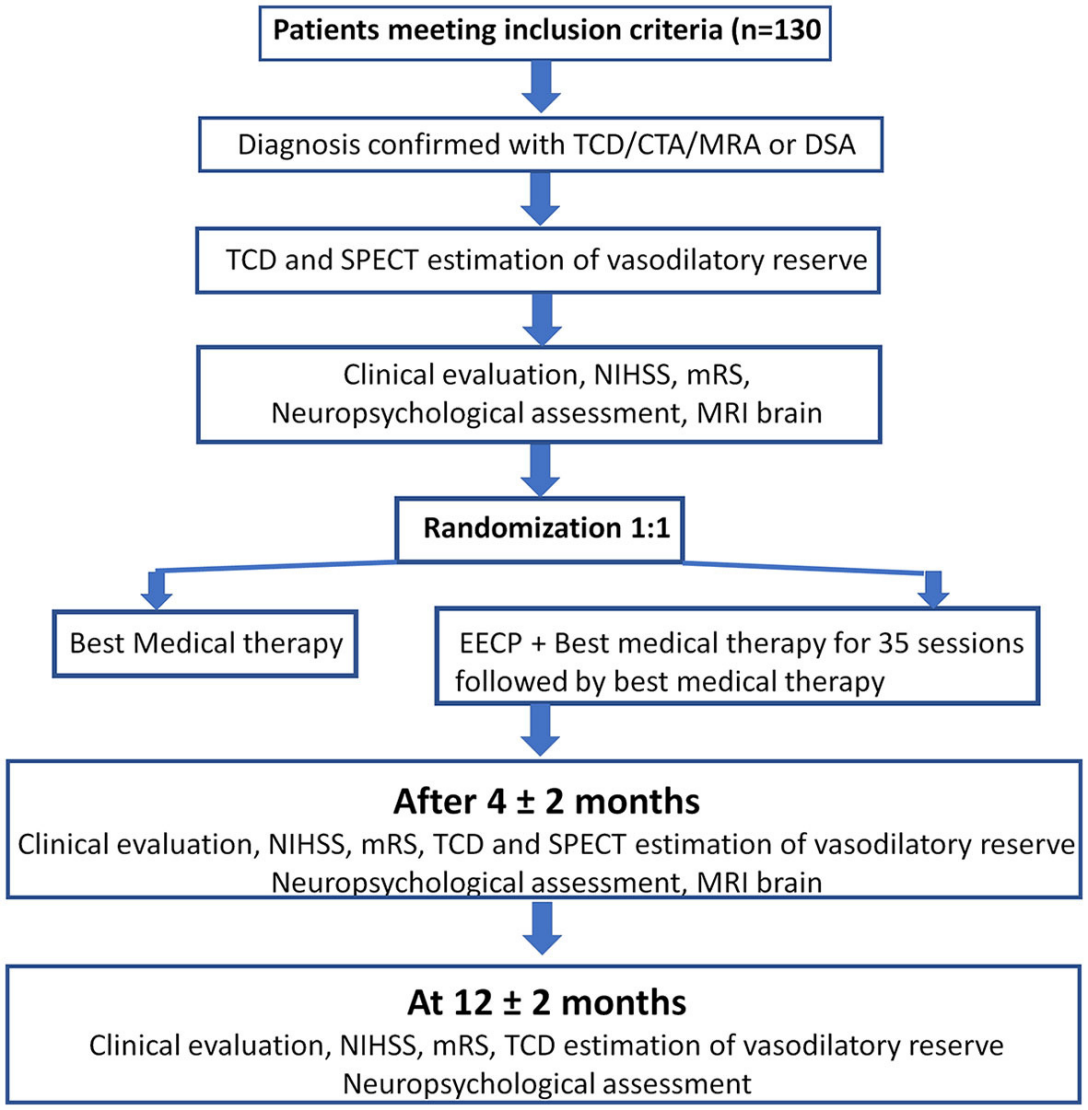
Schematic diagram of the workflow of the study.

This is a prospective, randomized, controlled, observer-blinded phase III trial. The study will be conducted at the National University Hospital, Singapore. The study would recruit a total of 130 participants. The primary objective will be assessed at 4 months after the initiation of treatment. Details of patient workflow are summarized in Figure 3.

### Patient population

Consecutive patients with symptomatic and severe ICAD involving intra-cranial ICA or MCA would be included in the study. We define “symptomatic” ICAD as the identification of a severe stenosis or occlusion of the index artery (by TCD and contrast CT angiography or digital subtraction angiography) in patients who present with symptoms attributable to that particular artery within the previous 3 months.

### Inclusion criteria

1. Patients with recent stroke/TIA and severe stenosis of intracranial ICA or MCA and impaired CVR within the previous 3 months (but not before 3 weeks) after acute stroke or TIA. This is to differentiate between patients with long-standing fixed stenosis from patients with the partially recanalized intracranial artery (masquerading as severe stenosis).
2. Age > 21 years
3. A modified Rankin score (MRS) of 3 or less.

### Exclusion criteria

1. Patients with atrial fibrillation/arrhythmias.
2. Within 2 weeks of cardiac catheterization or arterial puncture at the femoral puncture site.
3. Decompensated heart failure (NYHA class 3 or 4).
4. Left ventricular ejection fraction (EF) < 30%.
5. Moderate or severe aortic regurgitation.
6. Persistent and uncontrolled hypertension (BP persistently > 160/100 mmHg).
7. Bleeding diathesis.
8. Active thrombophlebitis/venous disease of lower limbs.
9. Severe lower extremity vaso-occlusive disease.
10. Presence of a documented aortic aneurysm/dissection requiring surgical repair.
11. Pregnancy.

## Baseline evaluation

### Clinical

Patients eligible for the study will be evaluated for their neurological deficits and functional status by using the National Institute of Health Scale Score (NIHSS) and MRS, respectively.

### Diagnostic TCD

Complete TCD evaluation would be performed with a 2 MHz pulse wave Doppler probe using the commercially available TCD machine. Intracranial stenosis would be diagnosed according to the established as well as locally validated criteria (47, 48). ICAD would be considered severe (>70%) according to the velocity criteria and presence of “blunted” flow spectra in the arterial segment distal to the stenosis (49). Tandem lesions in a single arterial tree (intracranial ICA and MCA, proximal MCA and distal MCA, extracranial ICA and ipsilateral intracranial ICA, or extracranial ICA and ipsilateral MCA) if associated with blunted flow spectra in distal MCA would also qualify the criteria for >70% stenosis.

### TCD emboli monitoring

All patients would undergo continuous TCD monitoring for 40 min to detect spontaneous microemboli in the index intracranial artery distal to the stenosis. It would be performed using Spencer's TCD head frame.

### TCD assessment of cerebral vasodilatory reserve

Cerebral autoregulation enables a constant cerebral blood flow (CBF) over a wide range of systemic blood pressure by varying the diameter of the intracranial arterioles (50). This may fail in some patients with severe ICAD. TCD can measure vasomotor reactivity (VMR) by assessment of flow velocities in response to increasing carbon dioxide levels. This can be performed by a simple breath-holding test and calculating the so-called breath holding index (BHI) (51). BHI is calculated as the relative increase in the MFV during breath holding divided by the time of apnea in seconds. In normal persons, the BHI amounts to 1.2 ± 0.6. An impaired BHI can help to identify patients at higher risk of recurrent cerebral ischemia (29, 52). This test will also identify patients with intracranial steal phenomenon (reversed Robin Hood syndrome) (53). This method has been validated in our previous study for selecting patients eligible for revascularization among patients with severe and symptomatic ICAD (29, 54). Based upon our previous experience, we will be using 0.69 as the cutoff for TCD VMR. A value of <0.69 would define impaired CVR. We will calculate the steal magnitude according to the previously published criteria (29).

### Magnetic resonance imaging

All patients would undergo the standard MRI of the brain to exclude a new ischemic stroke (diffusion and absolute diffusion coefficient), subacute stroke (fluid-attenuated inversion recovery-FLAIR), and intracranial hemorrhage (gradient echo). Time-of-flight MR angiography would be performed to document ICAD. In addition, all MRI scans would include arterial spin labeling (ASL) to assess regional CBF. ASL is a well-established MRI method for assessing cerebral perfusion in a quantitative manner, yielding values in ml blood/100 g tissue/min (55). ASL offers the benefit as it enables longitudinal assessment of perfusion in a vascular territory. It does not require the use of gadolinium-based contrast agents. This makes ASL an attractive candidate for monitoring disease progression or treatment response in various cerebrovascular disorders (56, 57). We will be using selective pseudocontinuous ASL with a circular labeling spot to evaluate regional CBF.

### Acetazolamide-challenged single photon emission computerized tomography

Acetazolamide is a potent cerebral vasodilator and is increasingly being used to assess the hemodynamic reserve in the brain (58). Intravenously administered acetazolamide induces vasodilatation of cerebral vasculature, increasing both CBF and cerebral blood volume (CBV). The maximum response is noted ~25 min after intravenous injection. Decreased reactivity to acetazolamide represents a reduced CVR. Patients with severe arterial stenosis who show little or no improvement in CBF in response to acetazolamide are known to carry a high clinical risk of recurrent cerebral ischemia or infarction after arterial occlusion, which is believed to be due to an insufficient supply of physiologic collateral vessels (15, 59, 60).

SPECT is used for the assessment of CVR by comparing the images before and after the infusion of acetazolamide. CVR is preserved and blood flow generally increases in response to acetazolamide in most cases where the perfusion pressure is normal and sufficient collateral vessels are established. However, when the collaterals are inadequate, the CVR decreases, and blood flow does not increase or may even decrease after administration of acetazolamide. Thus, brain SPECT with acetazolamide challenge can identify patients with reduced CVR. Hirano et al. (61) reported that patients with reduced CVR had significantly lower CBF values and higher CBV/CBF ratios compared to patients with normal acetazolamide reactivity. Reduced CVR on SPECT corresponds to enhanced oxygen extraction fraction (OEF) and represents stage II hemodynamic failure as determined with positive emission tomography (PET) studies (58).

Positron emission tomography (PET) is considered the gold standard for estimating CVR. Using oxygen-15 (O-15) labeled radiotracers, PET is able to give estimates of cerebral perfusion and hemodynamic parameters and allows the quantitative determination of the degree of hemodynamic compromise in patients with occlusive cerebral arterial disease. It was used to select patients for STA-MCA bypass surgery in the COSS trial (28). However, PET cannot be applied routinely to stroke patients because of its cost and limited availability. Blood flow reserve assessed by brain perfusion SPECT correlates well with the OEF measured by PET. Furthermore, SPECT is widely available. Thus, we chose to do acetazolamide-challenged SPECT for evaluating brain perfusion and blood flow reserve. Based upon our previous experience, we will be using a net CVR magnitude of 6% or more (difference between baseline and acetazolamide-challenged SPECT) as the definition of vasodilatory failure on SPECT (29).

### Neurocognitive assessment

Vascular cognitive impairment (VCI) is one of the major sequelae of stroke and transient ischemic attack (TIA) with negative functional impact and an elevated risk for institutionalization, dependency, and death. Post-stroke VCI or dementia was reported in 24% of patients in a recent meta-analysis (62). Similarly, highly prevalent VCI (43%) has also been reported in Singaporean patients with non-disabling ischemic stroke (63). Of this population, 40% of patients had cognitive impairment without dementia, and a further 4% were diagnosed with dementia within 6 months of the index event. Interestingly, we demonstrated the impact of STA-MCA bypass surgery on neurocognitive parameters in patients with severe steno-occlusive disease of the intracranial internal carotid artery or MCA (64). We hypothesize that by improving cerebral hemodynamics, EECP treatment would improve cerebral hemodynamics as well as cognition. We will administer cognitive measures among patients recruited in this study at the baseline and follow-up. Additionally, to evaluate whether the effects of EECP are sustained, we will assess cognitive functions 1 year after completing EECP sessions. The measures would include brief cognitive screening tests (Montreal Cognitive Assessment and Mini-Mental State Examination) as well as a 30-min protocol for neuropsychological evaluation [Symbol-Digit Modalities Test (SDMT), Trail Making Test, Controlled Oral Word Association Test, Animal Naming, and the Hopkins Verbal Learning Test-Revised]. The formal neuropsychological battery employed in this study has been validated locally for Singaporean elderly people (65).

## Randomization

Patients would be randomized (1:1) by picking the lot from a box, prepared in advance. The included subjects (*n* = 130) will be randomly divided into EECP plus best medical therapy (*n* = 65) and best medical therapy (*n* = 65) groups according to a random draw of lots.

## Intervention

Patients randomized to the EECP plus best medical therapy will undergo 35 sessions (1 h per day, 5 days a week for 7 weeks), while the best medical therapy arm will receive the treatment according to the current treatment guidelines (66).

## Follow-up assessments

All patients would be followed up for a period of 1 year. An overview of the follow-up plan is represented in Figure 1. Briefly, the evaluations performed at visit 2 and visit 3 would be as explained as follows.

**Visit- 2** would be after 4 ± 2 months from the time of randomization. Cerebral ischemic events, if any, would be recorded. In addition to the clinical evaluation, all study participants would undergo TCD vasodilatory reserve, MRI (with ASL), acetazolamide-challenged SPECT, and a detailed neuropsychological assessment.

**Visit-3** would be after 12 ± 2 months after the randomization. In addition to the clinical evaluation, all study participants would undergo TCD vasodilatory reserve and detailed neuropsychological assessment.

## Case example

**Figure 4:**
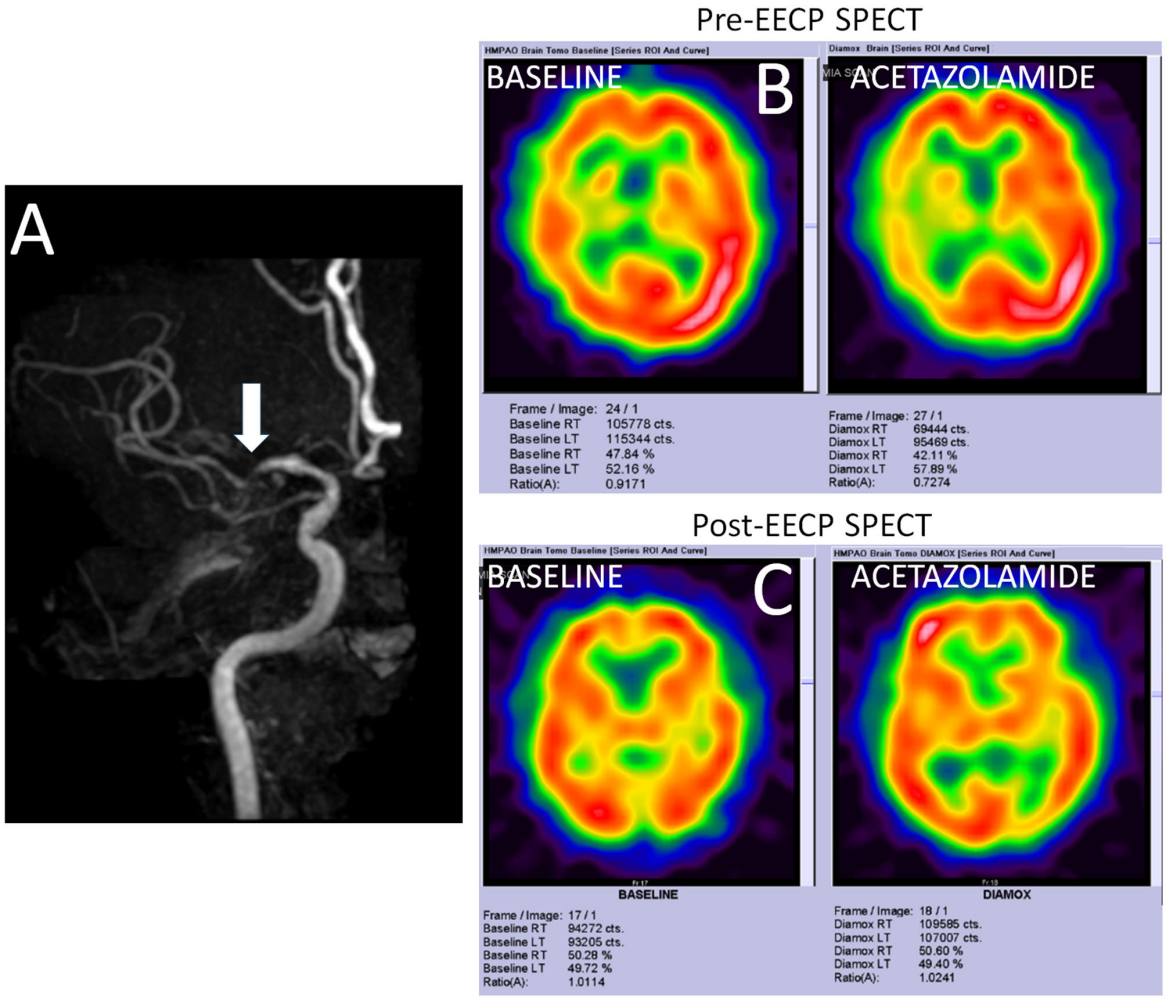
Imaging findings in a study participant. **(A)** Shows the time-of-flight MR angiography in a patient with severe stenosis of right middle cerebral artery (MCA). **(B)** Shows the SPECT findings at the baseline and after acetazolamide challenge. While only mildly reduced metabolic perfusion is noted in the right MCA territory at rest, acetazolamide challenge reveals a significantly impaired vasodilatory reserve (net perfusion deficit 11.46%). **(C)** Shows normalization of the metabolic perfusion at the baseline as well as after acetazolamide challenged SPECT.

A 52-year-old Chinese man presented with recurrent and transient episodes of left-sided weakness for the past 3 months. Each episode lasted for nearly 15 min. His cardiovascular risk factors included hypertension and dyslipidemia. Neurological examination revealed mild left-sided residual weakness. MRI of the brain revealed old infarcts in both anterior and posterior watershed regions. MR angiography revealed severe stenosis of right MCA (Figure 4A). TCD showed blunted Doppler spectra in the distal right MCA and an exhausted vasodilatory reserve during the hypercapnoeic challenge. An impaired vasodilatory reserve SPECT (net perfusion deficit 11.46%) in the right MCA territory was noted on acetazolamide-challenged SPECT (Figure 4B). The MMSE score was 19 points. He underwent uneventful 35 sessions of EECP therapy. At 2 months after completion of EECP therapy, his TCD showed normal vasodilatory reserve on TCD as well as acetazolamide-challenged SPECT (Figure 4C). Interestingly, his MMSE score improved to 24 points. He has remained asymptomatic during the past 1 year.

## Sample size estimates

Our previous study on the role of STA-MCA bypass in patients with inclusion criteria similar to the current proposal revealed that the surgical revascularization resulted in improving metabolic perfusion (on SPECT) by 6 points (SD 7.2) (29). This resulted in a nearly 32% absolute reduction in stroke recurrence during 27 months of follow-up. However, the bypass surgery is invasive, not applicable to all cases, and has surgery-related complications. We presume that short-term EECP would have a milder improvement in cerebral hemodynamics (at least an improvement of 4 points (SD 6.5). We expect that this improvement in cerebral hemodynamics by EECP would translate into at least a 10% absolute reduction in stroke recurrence (on top of the best medical therapy). For sample size calculation, a sample size of 60 in each group (120 in total) is sufficient to detect a mean difference of 4 units (with SD 6.5 units) in CVR improvement from the baseline between EECP and medical management group with 80% power and 5% significant level. We decided to recruit 65 patients per group (130 in total) to allow for treatment dropouts, withdrawal, and lack of follow-up.

## Statistical analyses

Statistical analysis of all study endpoints was carried out on an intention-to-treat basis. In the event of the loss of follow-up, patients were still included in the analysis for the duration in which they are observed.

All the demographic and baseline characteristics were analyzed descriptively. Frequency tables are presented for categorical variables, while mean with SD or median with range, whichever is more appropriate, is presented for numerical variables.

A two-sample *t*-test was used to compare the CVR improvement from the baseline between the EECP group and the medical management group if the normality assumption got satisfied for the CVR improvement; otherwise, the Mann–Whitney *U*-test was used. Linear regression will be carried out for relevant covariates.

The recurrence of a cerebral ischemic event was compared by the chi-square test or Fisher's Exact test, whichever is appropriate, between the EECP group and the medical management group.

For evaluating the effect of EECP on cognitive performance, various cognitive domain scores (z-scores) were calculated.

Using the mean and SD of the baseline values of the whole sample, controlling the significant baseline characteristic factors, analysis of covariance was employed to compare the mean difference of the change scores from the baseline to follow-up of the cognitive domains and individual subtest scores between real EECP and sham-EECP patients. In addition, paired *t*-tests were conducted to compare the mean within-group difference in cognition before and after EECP treatment.

We present the baseline characteristic of the 83 patients recruited in the study (Table 1).

**Table 1:** Baseline characteristics of the recruited patients (*n* = 83).

**Variable**	**Best medical therapy (*n* = 41)**	**EECP + best medical therapy (*n* = 42)**	***p*-value**
Mean age (range) in yrs	57 (27–73)	55 (26–75)	0.512
Male gender, *n* (%)	24 (58.5)	29 (69.1)	0.430
Diabetes mellitus *n* (%)	17 (41.5)	13 (30.9)	0.712
Hypertension *n* (%)	22 (53.7)	27 (64.3)	0.452
Ischemic heart disease *n* (%)	3 (7.3)	3 (7.0)	0.613
Dyslipidemia *n* (%)	9 (21.9)	11 (26.2)	0.168
TIA *n* (%)	18 (43.9)	17 (40.5)	0.382
Stroke *n* (%)	23 (56.1)	25 (59.5)	0.561
MCA stenosis *n* (%)	29 (70.7)	31 (73.8)	0.361
Intracranial ICA stenosis *n* (%)	12 (26.3)	11 (26.2)	0.571
BHI on affected side (range)	0.14 (−0.23 to 0.41)	0.08 (−0.10 to 0.51)	0.628
% Net perfusion deficit on acetazolamide challenged SPECT (range)	9 (6 to 15)	11 (6 to 27)	0.317

## Discussion

Currently, the best medical therapy remains the most evidence-based therapeutic option for secondary prevention of cerebral ischemic events for symptomatic patients with severe steno-occlusive disease of intracranial ICA or MCA. Although intracranial stenosis and STA-MCA bypass surgery are performed in carefully selected patients, both modalities are associated with unacceptable peri-procedural risks. In this clinical trial, we aimed to evaluate the impact of EECP treatment on improving CVR (and reducing stroke recurrence) in patients with severe steno-occlusive disease of ICA or MCA. This non-invasive novel therapeutic intervention may help a larger number of patients to prevent ischemic events and reduce the stroke burden.

## Ethics statement

The studies involving human participants were reviewed and approved by the Domain Specific Review Board (DSRB; Ref. 2018/00040). The patients/participants provided their written informed consent to participate in this study. Written informed consent was obtained from the individual(s) for the publication of any potentially identifiable images or data included in this article.

## Author contributions

VS, AS, HT, PL, and AG contributed to the conception and design of the study. LW, JC, and VS organized the database. BT, VS, and LY performed the statistical analysis. VS and AS wrote the first draft of the manuscript. BC, JH, DT, MJ, YG, SS, CK, ZK, SP, and HT contributed toward patient recruitment and performed a critical review of this manuscript. All authors contributed to manuscript revision, read, and approved the submitted version.
